# Privileged Precarity: How the Mobile Middle Class Leverage Housing Insecurity as Labour Market Strategy

**DOI:** 10.1111/1468-4446.70118

**Published:** 2026-04-16

**Authors:** Tim White

**Affiliations:** ^1^ King's College London London UK; ^2^ London School of Economics and Political Science London UK

**Keywords:** class, housing, inequality, precarity, privilege, work

## Abstract

How does the ability to weather insecurity give some an upper‐hand over others? This paper examines the interrelationship between housing and labour market precarity among middle class young professionals. Drawing on interviews with residents of co‐living schemes—for‐profit shared housing where tenants are on temporary rental contracts—it explores how residential precarity is strategically leveraged in pursuit of tumultuous careers in the knowledge economy. I propose the concept of *privileged precarity* in order to interrogate this dynamic and the contradictory subjectivities emerging therefrom. Whereas precarity is experienced by working class and marginalised households as a state of oppression, we see how the privileged can harness it as an asset and resource. In particular, the precarious tenure relations of co‐living enabled participants to synchronise their lives with the rhythms of hyper‐competitive and insecure careers in the tech sector—quickly pivoting to new economic opportunities and interacting with work on the terms of its contingent availability. By way of conclusion, the paper calls attention to the socially stratifying potentials of privileged precarities, and reflects on the applicability of the concept to other social domains and relations of inequality.

## 1 Introduction

In recent years an extensive body of scholarship has emerged examining the intensifying labour market precarity experienced by young professionals in the knowledge economy (Lorey 2015; McRobbie 2016; Standing 2011). Temporary, part‐time and contract‐based forms of employment are increasingly the norm for some of the most prestigious and sought‐after career paths, from tech to the cultural industries. This has been bolstered by trends including the digitalisation of labour (Brown et al. 2010; Huws and Leys 2003) and the emergence of remote work (Woldoff and Litchfield 2021). Central to these discussions is the notion that—in contrast to earlier periods of labour relations where economic risk associated with the free market was borne by the nation‐state, governing institutions and employers—the contemporary period is characterised by a reallocation of said risk to individual workers (Busuttil 2021; Boltanski and Chiapello 2007).

Alongside this broader precaritisation and individualisation, locational flexibility is often expected of young professionals in the knowledge economy (Maslova 2022). This is sometimes at the behest of an employer, but frequent relocation is also associated with freelancers in pursuit of projects and professional networks (ibid). Whilst historically such self‐organisation, autonomy and mobility was linked to counter‐cultural movements and radical forms of artistic production, scholars have argued that today this perfectly accommodates the neoliberal mode of production, externalising responsibility and uncertainty onto the ‘flexible’ worker (Gill and Pratt 2008; Boltanski and Chiapello 2007).

Seldom discussed in the literature on these shifting labour relations is the question of housing. As do we all, this growing contingent of precarious knowledge economy workers need somewhere to live. How does the contemporary ‘flexible’ worker weather the flux and change inherent to their working lives through housing? In this paper I seek to provide answers to this question drawing on a study of ‘co‐living’ spaces—a form of corporate group rental housing emerging in major European and North American cities, where tenants are on temporary, insecure contracts. Through this case, I demonstrate how the increasingly multi‐sited and precarious nature of work in the knowledge economy is being supported by new forms of housing with similar attributes.

I develop the concept of ‘privileged precarity’ in order to make sense of this relationship and the contradictory subjectivities emerging therefrom. This describes how the middle class strategically leverage residential insecurity as a labour market strategy. Rather than experiencing precarity entirely as a state of oppression, participants often harnessed their ability to live precariously to their professional advantage. Whereas residential precarity is experienced by working class and marginalised households as disempowering and destabilising, we see how the privileged can harness it as an asset and resource. This is not, however, to say that participants are unafflicted by structural constraints and insecurities—particularly the inaccessibility and unaffordability of housing markets. They may leverage precarity to their advantage, but they are still affected by it. And in enabling young professionals to synchronise their lives with the rhythms of competitive and insecure labour markets, new terrains for extraction and exploitation emerge. I propose the paradox of privileged precarity as a way of capturing and teasing out these contradictions.

## 2 Privilege, Precarity and the Insecure Home‐Work Nexus

The rhythms of housing and labour markets have always been intimately intertwined. Throughout history, housing tenures and morphologies have sprung up in parallel with the emergence of industries, sectors and working patterns; and this has always been fertile ground for exploitation. Residential hotels and bedsits, for example, played a key role in the movement of migrants and farm workers to North American and European cities during the industrial revolution—providing basic, temporary accommodation close to hubs of industry (Groth 1999). Research on the link between precarious housing and labour markets in the contemporary period has focussed particularly on low‐income and marginalised groups. Sociological studies have, for example, drawn attention to the ‘double precarity’ arising from the combination of insecure employment and housing. Desmond and Gershenson's (2016) research on low‐income renters in the United States links the disruption caused by a forced move, such as an eviction, to a significant increase in the likelihood of job loss. Based on a large‐scale household survey in Australia, Bentley et al. (2019), further find that insecurely employed households are five times more likely to experience housing affordability stress. Arundel and Lennartz's (2020) analysis of household data in the Netherlands also reveals how those who are labour market outsiders are significantly more likely to face housing market exclusion in terms of housing affordability, equity and prospective asset accumulation. More broadly, feminist materialist approaches to social reproduction and labour have stressed the mutually constitutive nature of precarious working and living conditions (Meehan and Stauss 2015).

In housing studies, scholars have deployed the ‘housing pathways’ framework to examine the residential trajectories of young adults (Clapham et al. 2014). Structural macroeconomic changes (e.g., deregulation, financialization of housing) interact with individual circumstances (e.g., class, income, family) to produce unequal outcomes (Arundel and Ronald 2016). Some of these studies explicitly examine the relationship between (relative) privilege and housing insecurity (Fang and van Liempt 2021; Jin et al. 2023; Revington 2025). This research has established how socio‐economic background, family support and financial resources significantly influence young people's ability to navigate precarious housing markets. This includes middle class households opting for precarious (usually private rented or shared) housing in desirable urban locations to access career opportunities and amenities (Uyttebrouck 2025). Hochstenbach and Boterman (2015), for example, highlight how differential social and cultural capital are strategically mobilised by young adults to secure housing in Amsterdam. The authors demonstrate how seemingly unstable pathways can represent tactics for navigating housing markets—including sacrificing residential security (long‐term contracts) to live in desirable locations. ‘Housing pathways’ studies therefore reveal the combination of structural constraints and individual circumstances shaping housing decisions and outcomes; and how the ability to navigate precarity has socially stratifying effects via class, race, gender, and other axes of difference (Revington 2025; Uyttebrouck et al. 2025).

The mutually constitutive role of housing and labour in the competitive and prestigious yet precarious knowledge economy has, however, been subjected to less scrutiny—with a number of notable exceptions. This paper takes influence from Ferreri et al. ’s (2017) study of property guardianship, where residents temporarily live in vacant properties at a discounted rent. The authors draw parallels between this insecure form of tenancy and the precarity of residents in terms of labour relations. It is argued that property guardianship represents an economic coping strategy for young professionals in the creative industries. Similarly, this paper draws on residents' experience of co‐living to consider the interrelationship between labour and housing market flexibilisation. In another study, Bergan et al. (2021) argue that co‐living—the same housing model covered in this article—represents a ‘home culture’ of precarity. Drawing on the marketing discourses of co‐living companies in New York, the authors contend that co‐living ‘provides a spatial foothold for precarious economic logics’ (p.1), reflecting the micro‐scalar production of the economy (ibid). But the authors do not consider if and how these home cultures are embodied by residents themselves—a matter to which this paper attends. Moreover, this paper varies from these accounts in emphasis. I argue co‐living enables participants to leverage their ability to live precariously to their own economic advantage, with both exploitative and empowering potentials.

This argument connects to the wider sociological literature on class and elite industries. These studies focus particularly on the role of the ‘bank of mum and dad’ in allowing young professionals to participate and succeed in certain careers (Brook et al. 2020; Friedman and Laurison 2020; Filby 2025). Friedman and Laurison (2020), for example, highlight how familial gifting and inheritance enables participation in tumultuous yet prestigious jobs like acting and television. The authors document how such an ‘economic cushion’ shapes career paths, the agency of some workers ‘scaffolded by the resources of others’ (p.91). This ranges from the type of work or industry, to the particularities of creative self‐expression and risk‐taking within, to the ability to resist extractive and poorly compensated roles. For example, in the case of acting, Friedman and Laurison demonstrate how economic privilege enables fledgling actors to be ‘picky’, accepting roles that avoid them being type‐casted or those that dilute their CV, ultimately bolstering long‐term career prospects. They show how, conversely, working class households ‘select out’ to less precarious industries. Although not the central argument, having ‘access to subsidised and well‐located housing’ (p.93) forms part of this picture. Focussing on young professionals at United Nations offices in Geneva and Vienna, Mülli (2021) similarly argues that privilege (access to unpaid internships and social capital) enables entry into precarious work (short‐term contracts, insecurity) that ultimately leads to prestigious careers at the UN. In these cases and others, the connection to housing is often implicit, and where directly confronted it is invariably about the economic cushion of asset ownership. But privilege is also leveraged in other ways through housing, especially in the increasingly private rent‐dominated economies of Europe and North America (Howard et al. 2024).

Considerable academic ink has been spilt debating the concept of precarity. On the one hand, the term denotes material political‐economic shifts. It emerged to describe how processes of privatisation and deregulation have given rise to conditions of insecurity for a growing swathe of the population in high‐income countries (Standing 2011; Beck 2000). In response, scholars hurried to point out that precarity has long been ubiquitous in many places and amongst many social groups, including working class and migrant households (Neilson and Rossiter 2008). Yet more complicated is the question of how precarity manifests at the individual level. As human beings we are all precarious to a degree, requiring different levels of support and care at different points in our lives, but this precariousness is highly unequally distributed (Lorey 2009). Scholars have argued that there is no objective state of precariousness, only varying states or experiences thereof (Della Porta et al. 2015; Umney 2022). This is a condition that intersects in deeply complex ways with individuals' cultural and economic circumstances (Jankowski 2024). One's state or perception of precarity is, then, at once subjective, relational, differentiated and existential. Recent sociological literature on precarity has paid particularly close attention to the importance of a multi‐dimensional perspective (Jankowski 2024; Antonucci 2018). This is to say, it is not just having an insecure job in itself that is a problem, but precarity emerges when these employment relations intersect with, for example, a lack of state and family resources (Antonucci 2018). Jankowski (2024) helpfully theorises precarity as a ‘landscape’ to capture the combination of individual subjectivities and objective political‐economic conditions involved. This calls for research exploring the mutually constitutive nature of different social and economic realms, including housing and labour relations.

The aim of this paper is not, however, to wade into the extensive debates on what constitutes precarity or precariousness. I instead embrace the inherent ambiguities of the term, and its multi‐dimensional, spectral and relational nature, to explore the link between insecure labour and tenure relations amongst middle‐class young professionals. I do not intend to suggest that this is a form of precarity without struggle and difficulty, and that my participants are not themselves operating within significant structural constraints. Neither do I, on the other hand, seek to claim the precarity they experience is equivalent to that of those in positions of marginalisation and poverty (e.g., Desmond and Gershenson 2016; Brickell and Nowicki 2025). As the ‘housing pathways’ literature shows, the trajectories of young people trying to exit homelessness (Pearl et al. 2021) are profoundly different from those of ‘young talents’ or creative professionals in the knowledge economy (Jin et al. 2023). Instead, I believe the dichotomy of privileged precarity can help shed light on the contradictory subjectivities and evolving class dynamics at play; highlighting the tensions and interrelations between choice and agency, subjection and empowerment, autonomy and exploitation reflected in participants.

## 3 Case and Methods

This paper is based on a mixed methods study of the ‘co‐living’ sector. Co‐living is for‐profit group rental housing. Frequently driven by financialised actors seeking to maximise returns from real estate assets (Casier 2023; Uyttebrouck and Paccoud 2026; White 2026), co‐living schemes are characterised by a combination of small private units, flexible rental contracts, and communal spaces and amenities (Ronald et al. 2024). Co‐living is a precarious form of housing. Whilst the specifics vary by jurisdiction, temporary contracts and weak tenants' rights are universal features of schemes across Europe and North America. Yet co‐living is also prohibitively expensive for low‐income groups and certain households (e.g., those with children, older individuals) are actively excluded. Most co‐living spaces are explicitly marketed towards young professionals. With flexible tenancies, all‐inclusive services and community events, they are sold as perfectly accommodating the transitory and work‐intensive lives of knowledge economy workers (Bergan et al. 2021). In this sense, they rebrand residential precarity as an entrepreneurial resource (Harris and Nowicki 2018; Harris 2020). Indeed, Casier and Revington (2025) argue that co‐living firms actively ‘exploit the life course’ – taking advantage of the housing market constraints faced by young professional in order to extract profits. Co‐living firms often operate properties across multiple cities and countries, and allow residents (frequently referred to as ‘members’) to move flexibly between schemes in different locations (White 2024a).

This paper draws on 22 semi‐structured interviews with co‐living residents across a variety of European and North American cities (see Appendix 1). My selection of co‐living spaces and tenants was company‐led: I was interested in speaking to residents of the largest firms in Europe and North America, which operated across multiple cities and countries. Participants were recruited by contacting those leaving online reviews of co‐living companies on platforms including Facebook, Reddit and Instagram, and subsequently snowballing. Most of my participants were in their 20s or early 30s, university educated and white. 60% were male. This is broadly representative of the typical socio‐demographic profile of co‐living clientele according to industry surveys (Everything Coliving 2026). However, any such small qualitative study is inherently partial, and I do not seek to claim that the study captures everyone's experience of co‐living, which will vary considerably by subject, location and co‐living company.

Due to my reliance on snowballing, the bulk of interviewees were from three cities: Berlin, Austin and London. All three are major employment hubs experiencing significant inflows of young professionals, and each is in the midst of an acute rental housing crisis. Berlin is a majority renter city characterised by an inside‐outsider housing system: newcomers are often funnelled into the expensive and precarious furnished temporary apartment segment, which co‐living schemes form part of. London is an owner occupier‐dominated city with a lightly regulated, highly financialized private rental market offering weak tenant protections and facing widespread affordability pressures. Austin, the fastest growing metro area in the United States, has been subjected to a particularly aggressive wave of gentrification and house price inflation over the past decade. This owes in part to the rapid influx of employees working for newly relocated tech firms, and a highly speculative, private equity‐dominated multifamily market. The particularities of these housing systems significantly shaped outcomes for residents.

Interviewees were provided with an information sheet, informed consent and the right to withdraw. The interviews took place between 2019 and 2022, and ranged from 35 to 120 min. They were open‐ended and free‐flowing in structure, and I encouraged participants to guide the conversation in terms of ‘what they believed to be relevant’ (Bryman 2008, 268). I would start by inviting them to explain how they ended up in the co‐living space. This would lead to questions about their occupation, where they were from, etc. I would then shift the conversation towards their day‐to‐day experience of the space itself.

All interviews were transcribed by the author and anonymised; participants' names, workplace names, and identifying details are pseudonyms or alterations. Interview transcripts were inputted and organised using NVivo software. Here the coding was performed in three steps. Firstly, I scanned over all the material and developed a set of broad hierarchical categories such as ‘flexibility’, ‘precarity’ or ‘entrepreneur’. I then performed a rigorous, line‐by‐line coding process for each interview, during which many sub‐codes and sub‐sub‐codes formed beneath the broad hierarchical categories. As part of this process, I took various measures to stay close to the data and remain open to analytical directions. One such measure was to adopt phrases or terms (e.g., that aptly captured a theme or perspective) used by respondents as sub‐codes in and of themselves so as to centre the voice of participants (Charmaz 2006, 56). Finally, as the most significant or reoccurring codes emerged, I synthesised these into more conceptual codes such as ‘remote working mobility’ or ‘labour market contingency’. In all, this layered approach to coding ensured that I stayed as true to the data as possible whilst gradually developing my analytical take (ibid).

## 4 The Privileged Precariat

In mid‐2019, George—a software developer in his early 30s from Malmö, Sweden—received some good news. He had been offered a 6 month contract as a business intelligence developer for a tech start‐up newly headquartered in Berlin. George had been keen to move to Berlin for a while: he craved a change of scene, enjoyed the nightlife and had several close friends in the city. There was just one problem: accessing housing, and fast:

… you know how it is when you're new in a city and a country, it's hard to find a place to live. I was moving to Berlin and I needed something fast that was simple and that could get me the proper documents, so I can sort out my German paperwork….

There were few immediately available options in Berlin for expats such as himself, George explains, the housing market polarised between expensive short‐term rentals and seldom available long‐term contracts. After a brief online search he found something that accommodated his needs: a co‐living space in the Friedrichshain district that he could move into immediately, fully furnished and offering flexible terms:

You could just literally book a room and move into a furnished room… I knew that it was expensive, but then you don't have a choice, how am I supposed to find from another country an apartment with short notice? I got a job offer and had to move there in three weeks and then you're in a time crunch and then you just, you know, go for the easiest option.

Within weeks George had moved in, finding in the co‐living space a remarkable concentration of residents in precisely the same situation as himself: recently relocated ‘expats’ in the tech sector.

Axel, a 23‐year‐old start‐up founder from Moldova, had a parallel experience. His company had been accepted for a reputable business accelerator programme in London, and he and his colleague had to quickly relocate to the city for a period of 12 weeks. Once again, they struggled with finding somewhere to live and needed to minimise housing costs:

… it's super hard to find living space in London, and Airbnbs and hotels are super expensive, especially for such a company like us, cause we were pretty early stage. We didn't have lots of funding. We didn't have lots of money in the bank. So we were looking for different ways to bootstrap this experience….

The director of the accelerator programme suggested the pair look into a large co‐living space in northwest London where other tech entrepreneurs were staying, and they quickly booked a ‘twodio’:

… [the accelerator programme director told us] lots of other startups are moving there because it's super easy to just find a housing place in there and you can just move in, it's oriented to be long‐term and not short‐term, like Airbnbs for a couple of days. And then it also has some pretty okay prices.

Craig, a tech firm employee originally from Australia, had secured a job in Austin, Texas as part of an overseas ‘adventure’ and needed somewhere to live whilst he found his feet in the city:

I wanted to leave Australia and like go on a bit of an adventure and live overseas. And so I started by getting a job in the US. So I got an offer and all that stuff. And then after that I needed somewhere to live.

Craig found in co‐living an immediately available, all‐inclusive, non‐committal housing solution:

I guess the benefit of like the co‐living thing for me is that like it's fully furnished, it's short term… in my situation, like effectively immigrating as an expat to the US, it's like pretty much perfect. So I guess how did I end up here? I knew I wanted to live overseas. I didn't know where or like how, but yeah, this fit that need really well.

For George, Axel and Craig, then, co‐living provided the necessary residential setup for them to quickly pivot to economic opportunities in new locations. They had the economic resources and autonomy to embrace this flexible housing option, but were still funnelled into it by the structural constraints of inaccessible and unaffordable housing markets and labour market demands.

George, Axel and Craig embody the bulk of my research participants: mobile young professionals entering a new city for the first time. The most striking continuity, beyond them being largely white and often male, was profession. The vast majority worked in the ‘knowledge economy’. There was a particular preponderance of individuals who were either entrepreneurs in the tech sector seeking opportunities to expand their business, or employed by tech companies and travelling at their behest. Several residents commented on the remarkable levels of professional homogeneity in their co‐living spaces, including Austin‐based Craig who himself worked in tech: ‘… everybody worked in tech and stuff like that basically, so everybody was a developer or had like an eCommerce store that they worked for, something like that, or digital marketing like me’. George also observed that his Berlin co‐living space was dominated by individuals from particular industries, and even companies—explaining that half of the people in his building worked for the online fashion company Zalando:

It was full of expats. So people in similar situations who had just moved to the city and to the country… There were no Germans in that building and the whole building was this co‐living space… like half of the building were software engineers, literally. Like every other one. And like half of the building worked for Zalando, because half of the expats in Berlin work for Zalando. So it's [a] very specific crowd, literally three professions: software engineer, data analysts, maybe quality products, stuff like that.

Interviewees were also characterised by particular employment relations within these industries. Many were self‐employed, freelance or contract‐based workers in pursuit of future project opportunities. Others were working for fast‐moving tech companies, experiencing a high degree of uncertainty about their future employment. This echoes studies of the cultural and technology sectors more broadly: whilst they are aspirational and require high levels of education, and can offer high pay, they are also characterised by sporadic, uncertain and insecure incomes (McRobbie 2016). As I will explore, co‐living accommodated the rhythms of these working lives in a multiplicity of ways, enabling individuals to put themselves in an advantageous position in relation to labour markets.

Though interviewees embraced residential instability in pursuit of their goals, this was often within a broader context of economic security. Alexia, a London‐based marketing professional working for a newly established beauty brand, connected her decision to opt into co‐living with the fact that her family owned multiple houses in Portugal, explaining ‘I actually come from a family where we have a lot of houses back home in Portugal so I don't feel the need to buy a house…’. Life coach Lee had purchased a flat in London and was renting this out whilst he lived at the co‐living space. Fitness instructor David further observed of his fellow residents: ‘A lot of people in this complex probably own lots of houses, but they want to have the flexibility of living in a place like this and not be attached to anything’. Therefore, whilst pursuing tumultuous career paths, many interviewees evidently harboured privilege in terms of wealth and inheritance. Their decision to opt into this precarious housing form was undergirded by the ‘economic cushioning’ (Friedman and Laurison 2020) in other parts of their lives.

As the opening stories of George, Axel and Craig demonstrate, the demands and goals of individuals, and their socio‐economic backgrounds, intersect in complex ways with the reality of local housing markets (Clapham 2002). Interviewees' decision to move into a given co‐living space, and their day‐to‐day experiences therein, were considerably shaped by the housing alternatives on offer and local context—including the particularities of tenancy regulations, the composition of housing stock and demand pressures, to name but a few. As largely outsiders, whether from different parts of the same country or entirely different nations, most lacked knowledge of the local housing market. A study undertaken by Habyt, the leading co‐living brand in Europe and North America, found that 70% of residents were internationals relocating for study or work (Habyt 2023). Respondents were therefore frequently funnelled into co‐living by a combination of their need for temporary and flexible housing (in lieu with their professional lives), and their lack of knowledge about (potentially less expensive, more informal) options. This was particularly apparent in ‘insider‐outsider’ housing markets like Berlin, where newcomers rarely access ‘normal’, long‐term regulated tenancies—and are instead funnelled into a complex, semi‐informal system of subletting and temporary contracts (Aaron 2024). Co‐living companies actively exploit these insider‐outsider divides, positioning co‐living as a liberating solution to housing market constraints and exclusions, particularly those faced by young professionals (Casier and Revington 2025; White and Madden 2024; Uyttebrouck and Paccoud 2026).

The decision to live in a co‐living space is therefore never one of complete autonomy and freedom, and is shaped by considerable structural constraints that vary between places. This speaks to research on the transnational mobilities of ‘middling migrants’, which reveals how the relatively privileged status of those such as digital nomads (Hannonen 2020) and retirement migrants (Botterill, 2017) frequently obscures ‘financial insecurity, employment and visa obligations, or housing insecurity’ (Mancinelli and Germann Molz 2024, 190). For example, whilst they may leverage their passport, professional skills and networks to their benefit, ‘middling migrants’ often remain excluded from welfare regimes and important local institutions and infrastructures.

## 5 Privilege and Place

The relative privilege of participants was particularly evident in relation to place. Many interviewees were highly conscious of their class position, particularly when discussing the area surrounding their co‐living scheme. In some neighbourhoods, they reported a strong sense of socio‐spatial separation between those within and beyond the walls of the property. Shaun, a resident of a large co‐living space in a semi‐industrial part of northwest London, for example, described how the internal community was dominated by the middle class and unrepresentative of the wider neighbourhood:

I think pretty much everyone's very middle class as well… You've got no one there that works frontline services. Everyone's like managers or fintech. It's just not representative of the country. It is the middle class. And like the guy I shared with before, his mum and dad had paid for his rent… it's us and them, it's this weird little square with hipsters in the middle of a deprived area. And I feel really awful for that.

In contrast, when placed within already‐gentrified neighbourhoods, co‐living spaces were seen to fit the dominant socio‐demographic profile. As George, who lived in Friedrichshain—a Berlin neighbourhood he considered ‘hipster’—commented:

… these cool parts of cool neighbourhoods are already populated almost exclusively by young people in their twenties and thirties who are the same type of people that live in this [co‐living space]. And this one that I lived in was in a hip area of a hip neighbourhood… So it fit in with the whole crowd around there… that part of Friedrichshain is already pretty gentrified by the young middle‐class professionals.

The prohibitive cost of co‐living was seen as a particular driver of their class‐exclusivity. Although generally less expensive than a one‐bed apartment in a given area, co‐living is never the cheapest housing option. Firms bundle additional services and amenities into the rent, and the model promises exceptionally high per square metre yields for investors (White 2024b). Jane, who lived in the same London co‐living scheme as Shaun, remarked that locals would likely be shocked at how much they were paying:

… to a certain extent there was that divide [between co‐living space and neighbourhood]. I do agree now that [co‐living] is affordable living compared to flats in London. But I don't think it would be affordable for locals in Harlesden. And I think they [would] think it was a shocking amount of money to spend each month on rent.

Sally similarly suggested that her fellow residents were not those at the sharp end of the housing crisis, and could in fact afford anything they wanted:

… anyone who lives in a co‐living house is paying a premium for [having a] flexible and short term lease and having a furnished place. So you're not crunched for housing. I mean, it's serving people who are able to afford it, who could then afford anything they want honestly <laugh>.

There was therefore a strong feeling amongst residents that co‐living caters to a distinctly middle class clientele. They are simultaneously spaces of insecurity and exclusivity.

Moreover, many co‐living firms administer a class‐exclusionary ‘filtering’ of prospective residents; explicitly targeting young professionals in the creative and tech sectors. For example, Germany‐based LifeX promises landlords ‘Our tenants are highly educated professionals with high income’ (LifeX 2020). Access is often mediated through an application process, with questions like ‘what is your greatest passion in life?’ (Tribe Co‐living 2019) and requesting links to social media pages. Gaining entry to these spaces is therefore frequently contingent on certain class credentials. However, I do not seek to underplay the considerable range of class positions amongst interviewees, or claim that they fit within rigid socio‐economic categorisations. This is not a homogenous group universally free of economic struggle. And as the pathways of George, Axel and Craig suggest, co‐living is not necessarily a first choice.

Social privileges beyond class were also evident, albeit seldom explicitly acknowledged by participants. As Massey (1993) argues, the ability to engage in certain types of movement is unevenly distributed and socially differentiated. In particular, as discussed across the mobilities literature (Benson 2013), mobility is highly gendered and racialised, and the case of co‐living is no exception. The vast majority of participants were white and university educated. Their freedom to move from one location to another must be juxtaposed with the immobility of other social groups, particularly that of migrants resulting from bordering practices, reflecting unequal global hierarchies (Massey 1993). The clientele in co‐living spaces are framed not as migrants but ‘expats’ (Cranston 2017; Kunz 2020). Furthermore, most participants did not have to worry about the risk of racism and stigmatisation that a new location or group of flatmates might bring, reflecting the relative freedoms awarded to white bodies (Sobande et al. 2021). 60% of participants were men, and implicit in many of their accounts was a sense of male privilege—harking back to the figure of the flaneur (Dreyer and McDowall 2012) free to explore unfettered by gendered risks and vulnerabilities. Therefore, whilst this paper focuses particularly on class, there are gendered and racialised intersections that require further exploration in cases of privileged precarity. In what follows, I look more closely at how co‐living is leveraged by the privileged precariat to their economic advantage.

## 6 Residential Infrastructures of the Privileged Precariat

This paper has hitherto explored how the lives of co‐living residents are characterised by a combination of precarity and privilege. Their careers are often marked by high levels of flux and insecurity. Yet they work within elite industries such as high technology, and enjoy a wider sense of economic security. As a housing form, co‐living spaces embody this same juxtaposition: they are tenure‐insecure but class‐exclusionary. With this in mind, in what follows I unpack how co‐living provides a form of enabling infrastructure for the privileged precariat, with both empowering and exploitative potentials.

### 6.1 Locational Opportunism

Given the uncertainty and change characterising interviewees' working lives, a recurring priority was avoiding the long‐term commitment to place usually associated with housing. The notion of flexibility was commonly invoked: the need to move out and onto something new should circumstances change. In offering a quickly accessible and non‐committal housing solution, co‐living was seen to accommodate this. Connor, a tech entrepreneur based in New York, detailed how his decision to move into the space was motivated by the flexible lease terms offered amidst a broader environment of uncertainty, accommodating his variable and unpredictable working life:

[I lived there] because I needed something flexible. I'm an entrepreneur and was at that time not sure whether I'd remain in NYC, have to move home with parents, or head out to the Bay Area. So the flexibility of the lease was the most important thing… The idea that you can easily sign a lease with no headaches, not have to buy furniture, and only stay for three or six months is very attractive, especially in this age of uncertainty.

Similarly, Hamza and Chrissy explained that co‐living offered a flexible, short‐term option for moving from place to place in parallel with the vicissitudes of their careers:

[A driver of co‐living is the] flexibility that comes with social mobility as well. So you know, if your contract ends with your job and you need to move, you can just pick up and move without too much problem (Chrissy, management consultant).

…so in June I ended my lease at the apartment that I was at for about three years. And my intention was to continue working remotely as long as possible. And while I did that, I did not want to sign up for another lease. I wanted to have a more flexible option where I could do something like one month in a city at a time (Hamza, technology firm employee).

We can see here how co‐living accommodates a form of non‐commitment to place supporting the need for residents to remain adaptable in their working lives. In particular, the flexibility of co‐living matched the episodic nature of their careers, allowing them to quickly pivot to new economic opportunities and interact with precarious work ‘on the terms of its contingent availability’ (Jankowski 2024, 716). The insecure tenure relations of co‐living, in this way, synchronise with the insecure labour relations of the ‘flexible’ worker, helping them serve the demand for adaptability and immediacy in their professions (Boltanski and Chiapello 2007). Due to the geographically distributed and short‐term nature of these opportunities, co‐living enables them to seize the moment.

Interviewees' relations to place, however, varied considerably. Several needed to be near hubs of industry, or in a particular location due to their employer. For interviewees like Shaun, Conor, Hamza and Chrissy, co‐living offered a relatively affordable and flexible way of accessing locationally‐specific labour markets. On the other side of the spectrum there were those who identified as remote workers or ‘digital nomads’ (Sciuva 2025), who leveraged co‐living spaces for their combined pursuit of career‐building and travel. These interviewees saw in co‐living the opportunity to sample different places whilst expanding their professional network. Co‐living was seen to offer the necessary supporting infrastructure for this nomadic pursuit in a multiplicity of ways. Digital marketing professional Calum, for example, argued that co‐living companies provide ‘jumping off points’ as both companies and workers become increasingly globalised, smoothening the process of transitioning from place to place:

They [co‐living companies] are essentially like ambassadors and jumping off points for a new kind of way of life, digital nomadism I guess. They're a way for people to kind of enter into any of these cities or communities effortlessly, facilitating this kind of globalisation that's not just companies globalising, but also workers globalising… And it removes the friction of travelling around the world and still working… I think for a lot of people, people who are digital nomads, one of the most difficult parts is just the logistics and finding a place and finding internet, that kind of stuff, so if these companies can become hubs that ease that process, then they're going to be an attractive service for people living that lifestyle, digital nomads.

As Calum's quote details, co‐living companies were perceived to play a role in streamlining the international movement of so‐called digital nomads, providing a range of facilities and institutional arrangements that ease the logistics of temporarily setting up one's working life in a new location.

For location‐independent interviewees, the physical mobility enabled by co‐living was routinely leveraged as an economic strategy. This often took the form of ‘geographical arbitrage’ (Hayes 2014; Mancinelli 2020), with residents moving to places with a lower cost of living in order to enhance their purchasing power and savings. In the US context, for example, co‐living companies facilitated movement from expensive coastal cities to more affordable locations within the country. As Craig, who moved from San Francisco to Austin via a co‐living company, put it:

…for me, crunching the numbers, if you move from somewhere else to Austin or to Texas in general, you're going to make like five to ten per cent more of your income because you're not paying tax. So I'm like, do I want to pay like three grand a month in San Francisco and pay a crapload of tax? Or do I want to move to Texas and like pay no income tax?

Other respondents leveraged co‐living to relocate from expensive European and North American cities to lower‐income countries, where their wages went further. Calum and Zaina, who moved from the US to co‐living spaces in Columbia and Mexico respectively, heralded the financial benefits of this move, yet were conscious of the potential socio‐economic disparities this bred:

To me, you know, since I can work remotely, I can save so much more money if I go somewhere lower income and it's equally if not more interesting to me, so… it was kind of international gentrification, it was a way for digital nomads, people who compared to Colombians are far wealthier. We were all pretty middle class by western standards, but obviously far wealthier than the average Colombian… I mean my savings like skyrocketed once I went to Colombia cause I don't even know how you would spend an American or British wage there. So saving a lot of money. Um, and at the same time zone, [I] got to learn Spanish, see a new country. So yeah, a lot of benefits (Calum, digital marketing professional).

Mexico City is having right now a huge influx of people from all over, living and moving there. Cause it is a great city and your money, especially if you're from the US, goes a lot further, especially if you're from a major city in the USA. Lots of locals I knew in Mexico city who have great jobs do really well, but like [compared to] an American, a good American salary, a good Mexican salary is not even close (Zaina, project manager).

For some interviewees, co‐living firms were not only facilitating these nomadic lifestyles, but shaping them. Several participants' plans were strongly influenced by the other locations offered by their company, particularly when they were paying a ‘membership’ fee. Craig and Connor, for example, explained that their next move would be based entirely on the listings of their co‐living firm.

These findings underline the ambivalent nature of the mobilities facilitated by co‐living. On the one hand, in a profoundly neo‐colonial way, these spaces facilitate the transfer of high‐paid young professionals to lower‐income countries, enabling them to harness their privilege, purchasing power and passport. Most framed the opportunities provided by co‐living in liberatory and enabling terms, wrapped into narratives of experimentation, adventure, adaptability and autonomous agency. Self‐described ‘nomad’ participants spoke of embracing an unsettled, untethered existence—allowing them to remain adaptable to new projects and rapidly shifting working conditions (Boltanski and Chiapello 2007). However, at the same time, as Craig and Zaina's experiences suggest, these mobilities are leveraged to navigate a challenging economic climate and stay afloat amidst the rising cost of living in US cities. We can again see how co‐living has the potential to facilitate a form of privileged precarity, simultaneously reflecting an empowered, adventurous existence and an insecure economic strategy.

### 6.2 Built‐In Social Capital

It was not just the flexibility and mobility enabled by co‐living that was seen to yield economic opportunities for participants. Also regularly emphasised was its potential to provide a form of guaranteed, built‐in social capital—a way of temporarily accessing a new set of social connections. As tech entrepreneur Richard put it:

I think it's really solving a core need for me as a remote worker and one who can travel and has a freedom of location, how do I find and build connections? How do I have a built in community no matter where I go of like‐minded individuals who share my same interests?

The perceived sociality of co‐living spaces was often linked to participants' professional pursuits; community framed in relatively instrumental and strategic terms. As design consultant Joe argued, given the concentration of fellow residents working in parallel sectors, social connections were themselves associated with networking opportunities and the potential for future projects:

The digital nomads, kind of like their intention is very much to network with other professionals, collaborate with other professionals… and even in [co‐living space] there's quite a lot of tech industry, IP consultancy, like industries where there's a lot of room for collaboration. And maybe that's part of that all‐inclusive service. It's like okay, I have this like networking which could in the end make me a lot of money or it could make me a big opportunity.

This sentiment aligns with research identifying the importance of networking and social connections amongst freelancers in the knowledge economy, which are seen to drive activity in service of project formation (Gregg and Lodato 2017). In this way, co‐living arguably harbours what Wittel (2001) refers to as ‘network sociality’. In contrast to conventional notions of community, this is a self‐reliant, responsibilised, entrepreneurial form of sociability: project‐based, temporary, purposeful, human capital‐building behaviour wherein relationships are viewed as ‘economic resources’. Such behaviours—associated particularly with freelancers in cultural industries and digital media—are geared towards income and autonomy in an economic climate rife with risk and competition (ibid).

Relatedly, some participants saw co‐living as an opportunity for social mobility: a way of surrounding themselves with a particular middle class, upwardly‐mobile social group. David, who said he came from a ‘lower class’ in Australia, felt that by being there he was transcending classes:

I didn't come from an upper class at all. I feel like I came from a lower class from Australia and then coming here, I think it's building me up. So I have the opportunity to come into… this sort of co‐living space, and I now have the capability to go into the upper classes based on surrounding myself with like‐minded people.

For many interviewees, then, co‐living spaces were perceived to offer a social environment closely linked to potential future economic opportunities. They sought to harness these socio‐demographically homogenous spaces as a stepping stone in their professional lives. Added to the locational opportunism and contingency they facilitate, this is another way in which co‐living spaces are implicated in producing the communicative, cooperative and gregarious subjects that the hyper‐competitive, precaritised knowledge economy demands (Boltanski and Chiapello 2007).

### 6.3 Officing

Finally, co‐living was seen to provide an internal convenience linked to participants' working lives. Co‐living companies offer a range of residential services and amenities that interviewees argued accommodated their demanding and unpredictable schedules. Several heralded the advantages of having everything immediately available and ‘built in’ to the spaces, especially given their long working hours and limited free time. Referring to the wealth of all‐inclusive services, Jane, a financial sector worker in a large London co‐living scheme, contended: ‘I generally work fairly long hours, so to be able to go home and have everything I need there, it's very convenient’. Similarly, Chrissy and Lee argued:

I think the motivator is the convenient lifestyle aspect. So people are very busy. They often work long hours… So co‐living gives us that convenience. All your events, all your friends in one place, all your bills taken care of in one payment (Chrissy, management consultant).

… it was nice to just have everything wrapped together. Like all the bills paid, and you're just paying a set amount every month. The little things like having a cleaner, so you haven't got to worry about the upkeep. You just turn up, pay your bills, and everything else is on your doorstep (Lee, life coach).

Tech employee Craig further mentioned how having everything immediately available under one roof enabled him to smoothly transplant his life and rapidly re‐establish his schedule in a new location:

… there's like all the benefits… Like it's short term, there's no commitment. It's fully furnished. Like there's salt, pepper, olive oil, shampoo, conditioner, like everything is here. Utilities, internet, Google fiber, 500 megabits a second. These are the things that are important to, like, nomads.

Therefore, from utilities, council tax and Wi‐Fi, to shampoo and furniture, to meeting new people, co‐living played a key role in sustaining the time‐poor, work‐intensive, contingent lives of residents. In this way, they can be seen to provide the residential infrastructure for what Humphry (2014) calls ‘officing’. This refers to the various forms of invisible labour that go into preparing and maintaining the conditions for work amongst nomadic and mobile labourers—from accessing reliable broadband to being constantly available and accessible for future projects. These demands, Humphry asserts, are inextricable from the ‘larger process of socio‐economic and institutional restructuring that underpin the rise of “anywhere, anytime” work’ (p.189).

In all, through co‐living, we see how participants leverage their ability to weather residential precarity and flux in pursuit of their careers. Co‐living enables interviewees to mobilise a range of resources—mobility, flexibility, community and convenience—to sustain and propel their working lives. These insecure, corporate shared housing arrangements provide a way for young professionals to synchronise their lives, both spatially and temporally, with competitive and flexibilised labour markets. In the knowledge economy of late neoliberalism, labour demands produce subjects that are enterprising, polyvalent, communicative, tech‐savvy and cooperative (Boltanski and Chiapello 2007)—it is these characteristics that allow them to traverse the world of precarious work (Karlsson 2024). For those who can afford it, co‐living assists in the enactment of these traits, enabling young professionals to contort their lives into harmony with the extreme demands and vicissitudes of contemporary industry (Boltanski and Chiapello 2007). They are thereby simultaneously spaces of subjection and empowerment, autonomy and exploitation.

## 7 Conclusion

This paper examines the interrelationship between housing and labour market precarity among middle class young professionals. It does so using the case of co‐living—for‐profit shared rental housing where tenants are on temporary, insecure contracts. Interviews with co‐living residents reveal how these housing arrangments feature in individual practices of adaption and negotiation, particularly among those in the tech sector. Co‐living spaces sustained interviewees' working lives by providing them with quick, non‐committal access to hubs of industry in certain locations. They also enabled location‐independent workers to traverse new places whilst whilst harnessing the built‐in networking potentials and practical affordances. In so doing, participants could better synchronise their lives with the rhythms of hypercompetitive and precarious labour markets.

The paper argues that co‐living spaces sustain a form of ‘privileged precarity’, whereby residential insecurity is strategically leveraged in pursuit of labour market opportunity. Participants were in a position of volatility in relation to housing, but interviews revealed how there were paradoxically professional advantages to this. In particular, the mobility and flexibility facilitated by co‐living enabled participants to hedge or protect themselves against the economic risk inherent to their working lives. Crucially, however, the type of precariousness experienced by respondents is not something they are entirely forced into (Lorey 2009). They are not lacking in agency like those in positions of extreme precarity (Desmond and Gershenson 2016). They are instead harnessing their ability to afford being precarious so as to put themselves in an advantageous economic position.

Yet for interviewees this was not a not a clear cut case of liberation. Participants also leveraged co‐living as an economic coping strategy in tumultuous times, their trajectories shaped by significant structural restraints—particularly the inaccessibility and unaffordability of housing, and the uncertainty of future work. We see how co‐living enables participants to better service the globalised corporations of today, propping up the risky speculative production activities and fluid labour markets upon which contemporary capital thrives (De Peuter et al. 2017). This makes them fresh sites of accumulation, extraction and exploitation. The paradox of privileged precarity is proposed as a way of grappling with these contradictions. On the one hand, interviewees are taking advantage of co‐living to empower and propel themselves. On the other, they enable young professionals to become optimised neoliberal subjects serving and normalising the excessive demands the contemporary (job) market.

Privileged precarities have socially stratifying potentials. The ability to weather risk and uncertainty is, of course, unequally distributed in the first place, and these dynamics are compounding. Those without a safety net must prioritise stable housing and work because the stakes are too high: a false move can be crippling. The need to avoid risk means some have to make choices (e.g., exploitative, low‐wage work) that further perpetuate their precarity, trapping them in cycles of insecurity. In contrast, the privileged can insulate themselves from risk and uncertainty, and, as we see in this paper, even leverage it to their advantage. This enables them to strengthen their long‐term prospects by weathering instability in the now. Privileged precarities are therefore likely to perpetuate deep‐set social inequalities, allowing those with greater resources to gain yet more of an upper hand whilst normalising and entrenching precarity for those without (Harris 2020). The infrastructures that sustain privileged precarities, like co‐living spaces, are directly implicated in this social stratification. Therefore, whilst this paper focuses on the internal dynamics thereof, large‐scale studies of privileged precarities are welcomed. In particular, the case calls for research that better bridges the mutually constitutive hierarchies embedded within housing and labour markets (see Arundel and Lennartz 2020).

This housing manifestation of unequal opportunity has parallels with other forms of inequality discussed across the sociological literature, and the notion of privileged precarity has applicability across a range of social domains. This includes the role of parental wealth and inheritance gifting in allowing young professionals to stay on precarious labour market tightropes, for example, providing them with housing in proximity to specific hubs of industry (e.g., television) (Friedman and Laurison 2020). It also has utility for examining how race, social class, and immigration status intersect. For example, how stigmatising assumptions unsettle the relative privilege of middle class, yet racialised, individuals (Browne 2024). It further speaks to how certain types of work can be experienced differently within and between social groups. For example, Guthman (2017) contrasts white university‐educated young professionals volunteering on organic farms with migrant farmworkers in the same role: the latter do not benefit from the social capital or recognition this work brings, and are subject to an entirely different set of power relations and working conditions. Privileged precarities are painfully visible close to home in the field of academia, where insecure employment conditions and uncertain prospects are increasingly the norm, and where the ability to weather precarity early in one's career strongly determines chances of success long‐term. Across a wide range of social categories and economic sectors, interrogating the mutually constitutive nature of privilege and precarity can help bridge macro and meso‐level mechanics of social stratification.

However, the concept of privileged precarity is not without flaws and limitations. In focusing on how individuals strategically leverage precarity, we must not lose sight of the systemic and structural forces shaping their choices and experiences. Relatedly, as Butler (2016) has argued, in viewing precarity as a universal condition, there a risk of normalising it as an inescapable feature of modern life, rather than a specific, politically induced condition that can be challenged through institutional change. On a more conceptual level, in combining these two terms, each is at risk of losing its analytical precision. In order to mitigate these risks, it is important to situate a given case of privileged precarity firmly within its political‐economic context, identifying the structural forces that give rise to conditions of both privilege and precarity. Studies should also carefully consider how the experience of precarity and privilege varies between and within social groups to avoid flattening out important differences. Provided that such measures are taken, however, the notion of privileged precarity holds significant explanatory power.

## Funding

This research was made possible by funding from the Economic and Social Research Council, grant no. 2098616.

## Conflicts of Interest

The author declares no conflicts of interest.

## Data Availability

The author has nothing to report.

## Appendix 1 Participant Information. Participants' Names Have Been Pseudonymised and Any Identifying Details Removed

**Table:**

Pseudonym	Profession/sector	Age (if disclosed) or age bracket	Place(s) stayed in co‐living	Country/city of origin	Living alone?
Alexia	Digital marketing	24	London	Portugal	N
Sally	Architect	30	Austin	Elsewhere US	Y
Shaun	Technology firm	Early 20s	London	Northern England	Y
Craig	Technology firm	24	Austin	Australia	Y
Pierre	International student	Late 20s	Berlin	France	Y
David	Fitness instructor	Mid 20s	London	Australia	Y
Axel	Technology entrepreneur	23	London	Moldova	Y
Calum	Digital marketing	Mid 30s	Austin, Medellín, Manila	Elsewhere US	Y
Oliver	Green technology	37	London	Elsewhere UK	Y
Connor	Technology entrepreneur	Early 30s	New York	Elsewhere US	Y
Lee	Life coach	Late 30s	London	Elsewhere UK	Y
George	Technology firm	32	Berlin	Sweden	Y
Maude	International student	Mid 20s	Berlin	Australia	Y
Tom	Technology firm	Mid 30s	London	Elsewhere UK	Y
Hamza	Technology firm	Early 30s	Austin	Elsewhere US	Y
Richard	Technology firm	Early 30s	Austin	Elsewhere US	Y
Zaina	Project manager	36	Mexico City, Austin	Elsewhere US	Y
Sarah	Remote worker (sector not disclosed)	Mid 30s	Austin, Bali	Elsewhere US	Y
Pauline	Real estate	Mid 30s	San Francisco	Elsewhere US	Y
Jane	Fintech	54	London	Elsewhere UK	Y
Joe	Design consultancy	Late 20s	Manila, San Francisco	Texas	Y
Chrissy	Management consultancy	Late 20s	London	Elsewhere UK	Y
